# Evaluating the safety of investigational anti-Alzheimer’s drugs: a comprehensive meta-analysis in the past to current decade

**DOI:** 10.1590/1980-5764-DN-2025-0472

**Published:** 2026-08-24

**Authors:** Neelufar Shama Shaik, Harika Balya, Srinivasan Ramamurthy, Balaji Pandiyan, Joel Mart Elias, Suvarnalakshmi Gunturu

**Affiliations:** 1 Scient Institute of Pharmacy, Department of Pharmacognosy, Ibrahimpatnam, Telangana, India.; 2 Fujairah University, Fujairah, College of Dentistry & Health Sciences, Department of Pharmacy, UAE.; 3 Vels Institute of Science, Technology and Advanced Studies (VISTAS), School of Pharmaceutical Sciences, Department of Pharmacology, Chennai, Tamil Nadu, India.; 4 The Oxford College of Pharmacy, Department of Pharmaceutical Chemistry, Bengaluru, Karnataka, India.

**Keywords:** Alzheimer disease, Drug-related side effects and adverse reactions, Meta-analysis, Pharmacovigilance, Antibodies, Monoclonal, Efeitos colaterais e reações adversas relacionados a medicamentos, Metanálise, Farmacovigilância, Anticorpos, Monoclonais

## Abstract

Alzheimer’s disease is a progressive illness that results in the degeneration of neurons with considerable burden. The pharmacological treatments for Alzheimer’s disease include cholinesterase inhibitors, monoclonal antibodies, NMDA receptor antagonists despite having different adverse drug reactions (ADR) alongside the health benefits. Limited data exists for the complete safety evaluation across different classes of anti-Alzheimer’s drugs.

**Objective:**

The meta-analysis evaluated the severity, frequency and variability of ADR as a result of Alzheimer’s drugs to determine relative safety across various classes.

**Methods:**

This meta-analysis followed the Preferred Reporting Items for Systematic Reviews and Meta-Analyses (PRISMA) guidelines with studies derived from the United States National Library of Medicine (PubMed), Embase, Scopus and ClinicalTrials.gov that focus on randomized controlled trials (RCTs) and observational studies which detailed ADRs of anti-Alzheimer’s drugs. Jeffrey’s Amazing Statistics Program (JASP) software was used to conduct heterogeneity assessment (I2), risk ratio (RR) and publication bias analysis in addition to meta-regression.

**Results:**

31 studies were utilized. The studies with cholinesterase inhibitors exhibited more gastrointestinal ADRs and monoclonal antibodies exhibited amyloid-related imaging abnormalities (ARIA-H and E). The BACE1 inhibitors showed liver toxicity that led to increased patient drug discontinuation. A random-effects model was used for the analysis due to heterogeneity (I^2^>50%). The meta-regression analysis indicated drug class as a predictor for the type of ADR.

**Conclusion:**

The research demonstrates considerable variations in the safety of Alzheimer’s drugs that need tailored treatment supported by post-approval monitoring systems. The literature needs extended investigations of safety evaluation as well as practical treatment investigations in actual clinical settings. Unlike conventional reviews focusing solely on currently approved therapies, this study provides a cross-generational analysis of adverse drug reactions across both historical and investigational anti-Alzheimer’s agents, offering insights into mechanism-related safety patterns that may inform the development and clinical use of emerging therapies.

## RESUMO.

A doença de Alzheimer é uma enfermidade progressiva que resulta em degeneração de neurônios, com carga considerável. Os tratamentos farmacológicos para a doença de Alzheimer incluem inibidores da colinesterase, anticorpos monoclonais e antagonistas do receptor NMDA, embora apresentem diferentes reações adversas a medicamentos (RAM) em paralelo aos benefícios à saúde. Existem dados limitados para uma avaliação completa de segurança entre as diferentes classes de medicamentos anti-Alzheimer.

**Objetivo:**

A meta-análise avaliou a gravidade, a frequência e a variabilidade das RAM decorrentes do uso de medicamentos para Alzheimer, a fim de determinar a segurança relativa entre as diversas classes.

**Métodos:**

Esta meta-análise seguiu as diretrizes PRISMA (Preferred Reporting Items for Systematic Reviews and Meta-Analyses), com estudos obtidos na United States National Library of Medicine (PubMed), Embase, Scopus e ClinicalTrials.gov, focados em ensaios clínicos randomizados (ECR) e estudos observacionais que detalhavam as RAM de medicamentos anti-Alzheimer. O software Jeffrey’s Amazing Statistics Program (JASP) foi utilizado para realizar a avaliação de heterogeneidade (I2), razão de risco (RR) e análise de viés de publicação, além da metarregressão.

**Resultados:**

Foram utilizados 31 estudos. Os estudos com inibidores da colinesterase apresentaram mais RAM gastrointestinais, e os anticorpos monoclonais apresentaram anormalidades de imagem relacionadas à amiloide (ARIA-H e ARIA-E). Os inibidores de BACE1 mostraram toxicidade hepática, o que levou a um aumento da descontinuação do medicamento pelos pacientes. Devido à heterogeneidade (I^2^>50%), foi utilizado um modelo de efeitos aleatórios para a análise. A metarregressão indicou a classe de fármacos como preditora do tipo de RAM.

**Conclusão:**

A pesquisa demonstra variações consideráveis na segurança dos medicamentos para Alzheimer, o que exige tratamentos individualizados apoiados por sistemas de monitoramento pós-aprovação. A literatura requer investigações mais extensas de avaliação de segurança, bem como estudos práticos de tratamento em cenários clínicos reais. Diferentemente das revisões convencionais, que se concentram apenas nas terapias atualmente aprovadas, este estudo fornece uma análise transgeracional das reações adversas a medicamentos tanto em fármacos históricos quanto em agentes experimentais anti-Alzheimer, oferecendo insights sobre padrões de segurança relacionados ao mecanismo que podem orientar o desenvolvimento e o uso clínico de novos tratamentos.

## INTRODUCTION

 Alzheimer’s disease (AD) is a progressive neurodegenerative disorder that causes memory loss and other cognitive impairments, such as deterioration in language, learning, memory, visual-spatial abilities, reasoning, and behavior^1^ . The neurodegenerative disease appears as the primary cause of dementia since it was observed in 60–80% of worldwide dementia cases. The typical pharmaceutical AD treatment includes both cholinesterase inhibitors (ChEIs) and N-methyl-D-aspartate (NMDA) receptor antagonists as the primary classes of medication. Donepezil, rivastigmine, and galantamine inhibit acetylcholinesterase activity, thereby reducing the enzymatic breakdown of acetylcholine (ACh) and enhancing cholinergic neurotransmission^2^ . Memantine serves as a NMDA receptor antagonist that controls glutamatergic activity as the same mechanism was involved in membrane formation and synaptic plasticity. These medications reduce symptoms but they fail to stop AD progress^3^ . Scientists have developed monoclonal antibodies that target amyloid-beta (Aβ) plaques, which represent the main pathological condition in AD. Though the clinical efficacy is good, safety parameters regarding Aducanumab alongside lecanemab and donanemab that decrease Aβ burden remain unclear^4^ . Current research focuses on developing BACE1 inhibitors together with glutaminyl cyclase inhibitors along with neuroinflammatory modulators that specifically target different AD pathological processes^5^ . 

 Clinical studies have established hepatotoxicity as a side effect of the old AD medication tacrine which physicians stopped prescribing for treatment of AD year’s ago^6^ . Clinical trials linking monoclonal antibodies that target amyloid-β (Aβ) peptides and promote plaque clearance in the brain have demonstrated their ability to cause amyloid-related imaging abnormalities (ARIA), which involves brain edema and microhemorrhages^7^ . Some BACE1 inhibition trials showed worsening cognitive decline, which led to agent atabecestat being discontinued from development^8^ . Scientists have tested the neuroprotective properties of the anti-inflammatory drug hydroxychloroquine, yet need to prove its therapeutic value^9^ . 

 AD management requires an organized evaluation of adverse drug reactions (ADRs) and treatment efficacy and patterns of discontinuation of treatment across different drug classes because patients show diverse responses to therapy. Multiple clinical tests have assessed AD drugs for both effectiveness and safety, but earlier meta-analyses conducted studies to find individual drug groups or predefined end results^10^ . Very few studies present a consolidated analysis of adverse events together with dropout statistics in tandem with relative risk information for various pharmacological interventions. Novel immunological treatments together with disease-modifying strategies need assessment of safety levels against established symptomatic therapies^11^ . These gaps have prompted a meta-analysis which incorporates various drug classes to analyze ADRs that result from medical therapies and at what point patients discontinue their treatments. 

 This meta-analysis research provides essential information for clinical practice through drug selection guidance with the goal of performing a systematic assessment measuring the total ADRs which occur among various medication classes. The research investigates how safety issues affect patient decisions to stop their medications by utilizing the randomized controlled trial and observational studies as evidence to deliver a thorough and clinically applicable investigation of AD drug safety that will direct future research directions and treatment decision processes. 

## METHODS

### Literature search

 The investigation collected published data by adopting a thorough literature search for trials that evaluated adverse reactions of anti-Alzheimer’s drugs. The analysis covered the United States National Library of Medicine (PubMed), Embase, Web of Science and Scopus and ClinicalTrials.gov to include high-quality, peer-reviewed research. The research approach used keywords as search terms which related to AD with drug safety and adverse events, cholinesterase inhibitors, monoclonal antibodies and BACE1 inhibitors along with neuroprotective agents.

- no detailed BPSD symptoms prevalence; and
- no objective measure of caregiver burden.

### Inclusion criteria

 The research team adopted predefined selection parameters according to the Preferred Reporting Items for Systematic Reviews and Meta-Analyses (PRISMA) guidelines to choose studies.

- Randomized controlled trials (RCTs), prospective cohort studies and large retrospective studies which analyzed the adverse events of anti-Alzheimer’s drugs.
- Patients with confirmed AD of any stage and confirmed dementia with probable AD.
- Research analyzed Food and Drug Administration (FDA)-approved medications and investigational AD treatments.
- Studies with placebo or active control groups.

### Exclusion criteria

- The study lacked placebo or active control groups.
- The study failed to present separate information about ADR outcomes for its treatment and control groups.
- The included studies reported data from case reports as well as conference abstracts according to editorials and included animal studies with in vitro research.
- This research focused mainly on cognitive efficacy results, but did not provide sufficient information about safety data.

### Study selection

**Figure 1.**
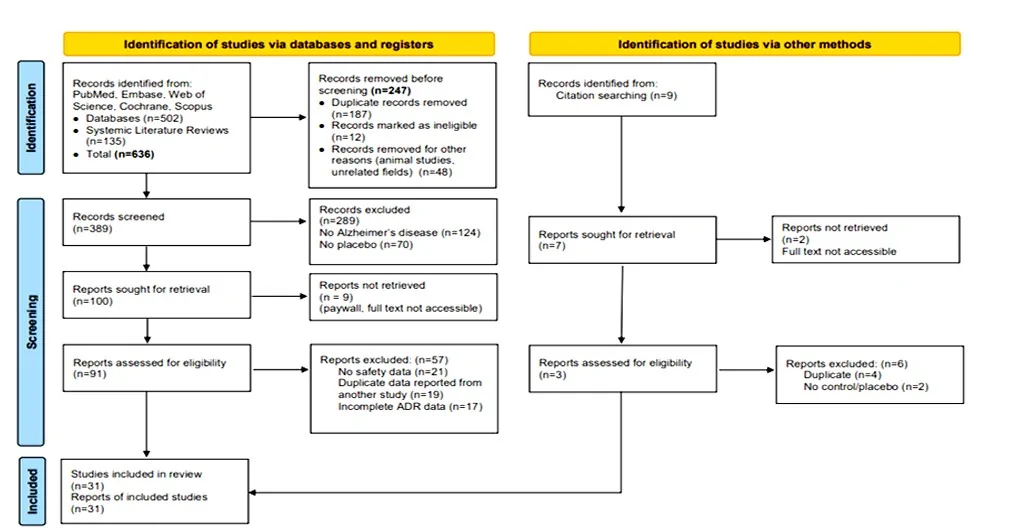
Steps included in the Preferred Reporting Items for Systematic Reviews and Meta-Analysis (PRISMA) 2020 flow diagram for study selection

Source: Self prepared.

 The selection of studies was conducted as per PRISMA 2020 guidelines^12^ . The literature research from 1990-till date yielded 636 studies, which included 502 electronic database articles and 134 systematic literature reviews. Screening of 187 duplicate records and 60 ineligible studies permitted initial review of 389 unique studies by observing their titles and abstracts.The screening led to the exclusion of 289 records where research was irrelevant (n=124) and case reports and editorials (n=95) and had no placebos or control groups (n=70). One hundred studies remained available for full-text eligibility assessment after completion of the retrieval process. The majority of analyzed studies (69) were eliminated because safety information was absent (n=21) or because duplicate data appeared in multiple studies (n=19), and because investigators had insufficient data on adverse events (n=17). The total selected pool contained 31 publications which participated in the quantitative meta-analysis. The steps included in the PRISMA 2020 flow diagram for study selection are given in Figure 1 ^13-42^. 

### Data extraction and quality assessment

 The extraction of relevant data from each included study was independently performed by two reviewers who used standard data collection forms. The collected data points included study identification data (researcher and publication year), research methodology, participant distributions between treatment groups and controls, follow-up and treatment duration, medication names along with drug classes, specified dosing regimens, administration route as well as ADR data that included their occurrence statistics, severity scales (mild, moderate, severe) with recorded treatment discontinuation. Data extraction discrepancies were settled by either achieving team consensus or involving a third senior reviewer in consultation. 

 The quality of the studies was determined by studying the statistical heterogeneity and potential publication bias through I^2^ analysis, Egger’s regression test and visual assessment of funnel plot asymmetry. Tests showing significant heterogeneity underwent subgroup and sensitivity analyses in order to determine their effect on overall findings. Continuity correction (0.5) was applied to the data to stabilize calculations. 

### Statistical analysis

 The statistical analyses were conducted using JASP2024 (Version 0.19.3) platform. The random-effects model served as the method to combine effect sizes because heterogeneous data was displayed across studies. The validity assessment depended on I^2^ statistics combined with Cochran’s Q test. Results showed that high heterogeneity levels above 50% indicated substantial differences between studies. Statistical results involved calculating risk ratios (RR) with risk differences (RD) while considering their respective 95% confidence intervals (CI) regarding dichotomous outcomes. Subgroup analysis were performed which evaluated drug class as well as dosage levels and therapeutic duration to examine what factors affected adverse drug reactions. Additionally, meta-regression analyses were conducted to examine potential moderators, such as age, baseline cognitive status, treatment duration, and dosage levels, in influencing adverse event risks. 

## RESULTS

 This meta-analysis examined ADRs and dropout rates along with heterogeneity between studies, and subgroup evaluation based on drug class categorization. Publication bias, meta-regression analysis and cumulative regression analyses were also performed. 

### Demographic data of included studies

 The meta-analysis incorporated 31 RCTs which focused on investigating the safety and adverse effects of anti-Alzheimer’s drugs through evaluation of 16,310 participants who received placebo/comparator drug treatment along with anti-Alzheimer’s drug (Supplementary Material Table S1 – available at  https://www.demneuropsy.org/wp-content/uploads/2026/05/DN-2025.0472-Supplementary-Material.docx). The studies included RCTs, double-blind placebo-controlled studies, and phase II/III multicenter studies. The majority of trials included mild to moderate AD patients, yet few studies examined patients with dementia that probably had AD. 

 Researchers examined different drug classes, acetylcholinesterase inhibitors (AChI), NMDA receptor antagonists, monoclonal antibodies (MCAs) targeting beta-amyloid, BACE1 inhibitors, vasodilators, antipsychotics, neuroprotective agents, and hormonal therapies. AChIs maintained are the most investigated drug class after monoclonal antibodies in the trials. Anti-amyloid monoclonal antibodies represent an important therapeutic strategy in AD, targeting amyloid-β peptides to promote their clearance and reduce amyloid plaque burden (Table 1). 

**Table 1.** Descriptive characteristics of included studies

Variable	Mean±SD
Total Sample Size	512±316
Mean Age of Participants (years)	71.5±7.3
Percentage of Male Participants	46.20%
Trial Duration (weeks)	38±28.5
Most Frequently Studied Drug Class	Acetylcholinesterase inhibitors
Route of Administration (Most Common)	Oral (67%) > Intravenous (28%) > Subcutaneous (5%)
Control Group Type	Placebo (81.5%) / Active Comparator (18.5%)

Source: Self prepared.

### Heterogeneity and effect size estimates

 Heterogeneity in the study data were analyzed using I^2^, τ ^2^ and RR with 95%CIs, also determining effect sizes and standard error. Heterogeneity between studies was significant because the I2 value, which was 93.6% (95%CI 90.997–98.59), is greater than 50% (p<0.001), which showed random differences in ADR rates were primarily due to different study populations along with drug regimens and methodological aspects. A random-effects model of analysis was chosen because the studies showed heterogeneity. 

**Figure 2.**
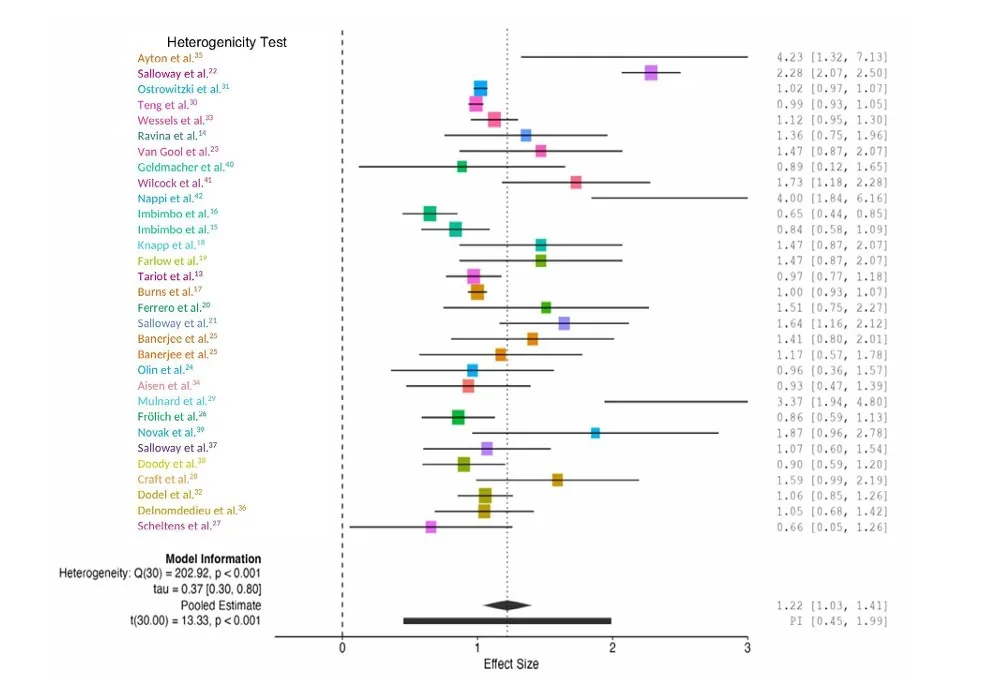
Forest plot showing heterogeneity.

Source: Self prepared.

 According to the pooled RR analysis, patients exposed to anti-AD drugs had a 22% higher likelihood (pooled RR=1.22) of reporting ADRs compared to patients in control groups (95%CI 1.03–1.40). Results from the prediction interval (PI 0.45–1.98) showed that the risk of ADRs in the treatment group varied significantly in different trials regardless of any observed significant differences. The variance in between trials (τ^2^=0.133; 95%CI 0.092–0.638) indicates major differences in the results of individual trials. In addition, Figure 2 presents clear evidence based on forest plot that the majority of studies demonstrate higher ADR frequencies in the drug-treated group versus the control group. Some research studies presented wider confidence interval ranges because of the varied sample sizes. 

### Publication bias analysis

**Figure 3.**
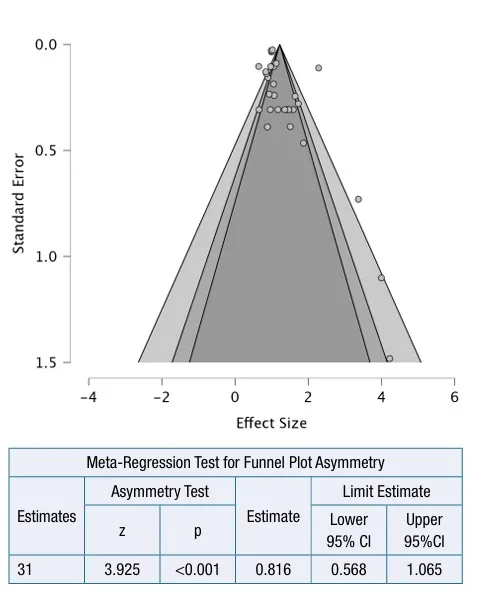
Funnel plot of the included studies.

Source: Self prepared.

 The assessment of publication bias was performed using Egger’s regression test combined with rank correlation test. Egger’s test showed statistical significance, p<0.001 (Z/t Statistic: 3.725) and the rank correlation test also indicated statistical significance, p<0.001 (Z/t Statistic: 0.421, asymmetry confirmed), suggesting potential publication bias or small-study effects. Figure 3 (funnel plot) demonstrated asymmetry, which may indicate potential publication bias or small-study effects. 

### Subgroup analysis by drug class

 The meta-regression analysis was conducted using subgroup analysis to identify how drug class might affect ADR risk rates. The ADR incidence showed significant variations between various pharmacological treatments, as demonstrated by the results (Table 2). The beta-amyloid-targeting drugs solanezumab, aducanumab, bapineuzumab, gantenerumab and crenezumab showed a significantly elevated ADR risk, as studies showed severe adverse events including amyloid-related imaging abnormalities (ARIA-H and ARIA-E) that affected the brain through vasogenic edema and microhemorrhages (Supplementary Material Table S2 – available at  https://www.demneuropsy.org/wp-content/uploads/2026/05/DN-2025.0472-Supplementary-Material.docx). Mon clonal antibody treatment of Alzheimer’s patients showed a high ADR risk, which led to a high dropout rate of 47.8% because of the numerous tolerability issues experienced with this therapy. 

**Table 2.** Subgroup analysis of risk of adverse drug reactions by drug class.

Drug class	Drugs Included	Risk ratio (95%CI)	Dropout rate (%)
Acetylcholinesterase Inhibitors (AChEIs)	Donepezil, Galantamine, Rivastigmine, Tacrine, Physostigmine	1.15 (0.97–1.38)	10.50
NMDA Receptor Antagonists	Memantine	1.03 (0.89–1.18)	8.90
Monoclonal Antibodies (Anti-Aβ Therapies)	Solanezumab, Aducanumab, Bapineuzumab, Gantenerumab, Crenezumab	1.42 (1.10–1.92)	47.80
BACE1 Inhibitors	Atabecestat, Lanabecestat	1.31 (0.98–1.69)	14.30
Adjunctive neuropsychiatric therapies	Carbamazepine, Mirtazapine	1.12 (0.94–1.34)	12.10
Hormonal Therapy	Estrogen, Insulin	1.07 (0.84–1.25)	10.20

Abbreviation: CI,confidence interval.

Source: Self prepared.

 The safety profile of memantine implies the activity as a therapeutic choice because of good retention rates of patients. Adjunctive agents used for neuropsychiatric symptom management in AD showed relatively lower ADR risks compared with monoclonal antibody therapies, but both categories had severe adverse effects that included cardiac problems and metabolic complications. Careful prescribing of MCAs should occur because these agents present both high ADR risks along with substantial dropout rates. 

### Effect of other parameters on risk of adverse drug reaction

 The meta-regression analysis study was conducted to determine if the study duration and dose levels affected ADR risks. The research data showed no clear link between higher dose and incidence of new adverse drug reactions (p=0.889), indicating that sole dose adjustments cannot prevent the adverse effects from happening. The study duration affected ADR risk negatively, but failed to show statistically significant changes (p=0.132) (Table 3). 

**Table 3.** Effect of dose, treatment duration on the dropout rate.

Parameter	Unstandardized	Standard error	Standardized	t	p-value
Dose	-1.132×10^-5^	7.966×10^-5^	-0.026	-0.142	0.889
Treatment duration (weeks)	0.010	0.006	0.366	1.615	0.132
Dropout rate	-0.003	0.003	-1.539	-1.191	0.257

Source: Self prepared.

 The analysis highlighted monoclonal antibody therapies as the crucial factor which contributed to ADR risk (p=0.001), thus confirming findings obtained through subgroup analysis. The study results indicate the pharmacological activity of monoclonal antibodies play the most important role in causing the reported adverse effects rather than treatment time or drug amount alone. 

**Figure 4.**
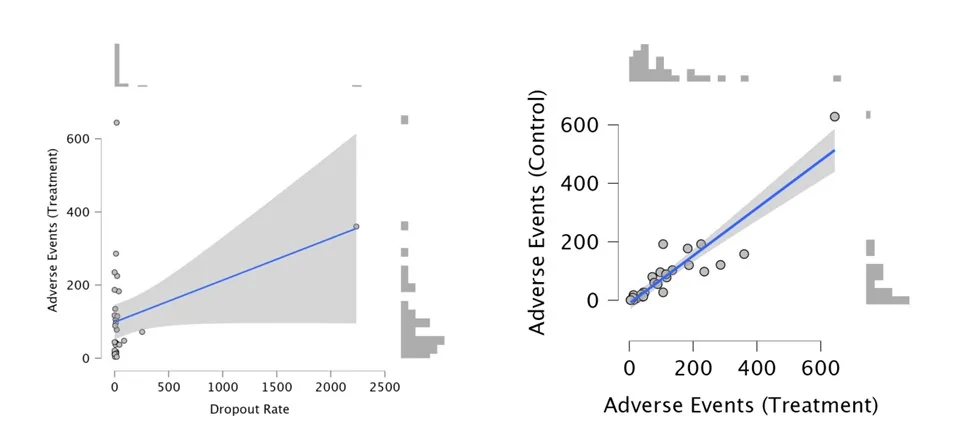
A. Relationship between dropout rate and adverse events in treatment group; B. Correlation between the adverse drug reactions in treatment and control group.

Source: Self prepared.

 The dropout rate analysis in clinical trials showed an upward trend when the number of adverse events exceeds in the treatment group. The results indicated that patients who encounter adverse events frequently choose to stop their medication treatment (Figure 4A). The data points extend further from each other at lower dropout rates because some adverse events do not lead to treatment disruption, but instead reflect patient tolerability and symptom severity. 

 The scatter plot in Figure 4B shows a regression line that indicates how treatment group ADRs relate to ADRs in the control (placebo) group, where a strong correlation represents the rates are higher in the treatment group. 

### Cumulative meta-analysis

 The researchers employed a cumulative meta-analysis to determine if ADR risk evolved during the study period. The results showed that the RR of ADRs were higher in older trials before 2010 (1.469; p<0.001); between 2010–2015 it was 1.29 (p-value 0.021) and subsequently decreased after 2015 (RR1.22; p-value 0.045). The current data shows that monitoring of the safety of drug formulations and patient management procedures have become more advanced through time. 

## DISCUSSION

 This meta-analysis conducted a detailed evaluation of ADRs that prevail within different AD pharmacological therapies. The research shows that drug classes generate diverse ADR profiles confirming safety matters which need proper management during clinical use. MCAs receive maximum attention in ADR treatment due to most frequent ARIA and cholinesterase inhibitors showing gastrointestinal (GI) and cardiovascular systems related to ADR. Existing systematic reviews show drug mechanisms together with patient characteristics significantly influence the variety of adverse effects^43^ . 

 Clinical data support that MCA treatment including aducanumab and bapineuzumab shows high rates of ARIA, since studies have shown that amyloid-targeting therapies showed a dose-dependent risk of ARIA. The drug mechanism of binding Aβ plaques during Aβ clearance triggers microvascular changes leading to cerebral edema and microhemorrhages, even though it slows a progression of AD^44^ . The risk rate for ARIA based on monoclonal antibody trials exceeds 90%, which demands rigorous clinical surveillance programs. The study showed bapineuzumab caused ARIA in 92.6% of patients, higher than the rates recorded for first-generation monoclonal antibodies, and also above recent ARIA incidence rates^45^ . Previous experimental data confirms that bapineuzumab and solanezumab treatment leads to substantial ARIA incidence rates similar to those found in our study^37,44^. Studies indicated that although aducanumab is second-generation MCA, it showed high ADR, which might be due to significant differences in trial population and patient selection criteria^15^ . The population-specific Apolipoprotein E4 (APOE4) genetic status appears to influence the number of ARIA cases reported differently between various research studies in this analysis. Recent monoclonal antibodies such as lecanemab and donanemab represent important advances in disease-modifying therapy for Alzheimer’s disease, and future meta-analyses incorporating these agents will provide further insights into their safety profiles. 

 The funnel plot showed asymmetry, which can also arise from other factors such as clinical heterogeneity, selective outcome reporting, differences in study size, or methodological variability across trials. Therefore, these findings should be interpreted cautiously. This variation might be due to the reason that we considered all minor ADRs also as potential side effects. The results from the meta-regression tests showed that trials appearing with fewer reported ADRs probably did under-reporting of these effects, thus affecting the overall analysis of drug safety. 

 Donepezil and galantamine showed different ADRs, since their predominant effects included gastrointestinal symptoms along with nausea, vomiting and diarrhea and abdominal pain. As per the mechanism of action, cholinesterase inhibitors increase gastrointestinal activity through their pharmacodynamic mechanism of enhancing cholinergic neurotransmission in the gastric and intestinal region^46^ . Multiple studies reported bradycardia and syncope as cardiovascular ADRs throughout the investigations, which supported previous studies that these drugs can worsen heart conditions in elderly patients with cardiovascular risks^47^ . Research conducted by Kobayashi et al. through a network meta-analysis confirmed donepezil showed higher gastrointestinal ADRs when compared to rivastigmine and galantamine within the cholinesterase inhibitor class^48^ . As there is a high level of heterogeneity implying complex ADR risk in various drug classes, subgroup analysis was conducted to study the patterns of ADR in different classes. 

 Research findings from this study confirmed that donepezil caused a significantly higher number of digestive side effects such as nausea and diarrhea, which confirms the existing concerns about its gastrointestinal tolerability. Previous studies have reported conflicting findings about whether cholinesterase inhibitor medications can cause cardiac bradycardia and syncope episodes due to their impact on vagal regulation, despite inconsistent statistical evidence^49^ . The literature shows conflicting results about the relationship between donepezil and AChEIs, with syncope and falls in older adults, since some studies show an increased risk while others report no statistical connection to placebo. Additional research is necessary to determine the precise cardiovascular risks related to these drugs due to the diversity of research designs and patient demographics. 

 Some drug classes show contradictory evidence regarding the severity of their associated ADRs based on recent evidence. Despite the general understanding that discontinuation rates due to adverse drug reactions (ADRs) within the cholinesterase inhibitor class are relatively low (9–15%), as reported in previous studies^50,51^, the present study demonstrated substantially higher rates. Moreover, gastrointestinal and neurological adverse effects were observed more frequently than those documented in earlier reports, which indicated overall dropout rates below 30%. 

 The results also indicated that benzodiazepines, which doctors often prescribe for neuropsychiatric symptoms in AD, produce substantial cognitive impairments, thus supporting concerns of ADR in additional therapies for this patient group^52^ . The research indicated higher severe adverse effect rates among AD patients using adjunctive neuropsychiatric therapies carbamazepine and mirtazapine, similarly to previous studies. Evidence has raised concerns about the use of benzodiazepine because long-term use elevated the risk of dementia^53^ . 

 The study examined ADR-related dropout rates extensively as a focus in immunotherapy trials. According to findings, studies with MCA showed higher discontinuation rates than other drug classes, which is in line with previous studies that support the tolerability of MCAs as a main limitation^44^ . The reported dropout rates exceed the numbers published in several past systematic reviews. The varying trial durations might explain this variation because more extended research often encounters higher participant discontinuation rates because of the cumulative impact of the ADRs. More investigation is needed to understand the emerging role of combination therapy because hydroxychloroquine showed promising results^23^ . 

 Our research findings hold significant clinical significance because of the differences that were observed in ADRs between drug classes. Physicians should conduct routine magnetic resonance imaging (MRI) screening tests together with individualized therapy adjustments when handling patient risk variables. Improved treatment compliance to cholinesterase inhibitors can result from developing new therapeutic delivery systems with enhanced tolerance, including transdermal drug patches. The evaluation of specific drug classes toward improving cognition requires healthcare professionals to carefully analyze their adverse effect profiles before prescribing treatments to individual patients. New-age personal treatment methods that utilize genetic profiles together with biological markers can predict which patients will adapt well to specific AD medications, thus producing better therapeutic results. 

 Future research needs to develop and enhance biomarkers which help identify patients at high risk for adverse effects specifically when receiving immunotherapy. Artificial intelligence, together with machine learning methods that evaluate genetic and biomarker data, will change the way personalized AD treatment is implemented through risk prediction of adverse events. Real-world patient data retrieved from electronic health record systems needs to be integrated into ongoing clinical trials for valid assessment of their findings. Literature must include extensive observational examinations of drug safety throughout continuous observation instead of clinical trials, while also studying various treatment approaches including drug remodeling and individualized medicine programs. The varied tolerability data from research requires expansive multi-center trials with numerous patient demographics to prove the current findings. 

### Limitations

- Bias was observed due to under-reporting of ADR cases (no ADR or few ADR). Clinical trials need to report their negative findings and non-significant results rigorously, because this practice minimizes bias within systematic reviews.
- Varying study methodologies, the duration and characteristics of enrolled patients, affects how patients tolerate their ADRs. Because unpublished data along with real-world observational studies were excluded from the study selection process, there could be a selection bias.
- Some of the pharmacological agents included in the analysis, such as tacrine, eptastigmine, and certain anti-amyloid monoclonal antibodies, are no longer widely used or have been discontinued during drug development. While this may limit the immediate clinical applicability of the findings, the inclusion of these therapies provides important insights into historical safety data and potential class-related adverse drug reaction patterns. Such information may contribute to a better understanding of mechanism-related adverse effects, and may inform the development and safety monitoring of newer therapeutic agents.
- However, recently approved therapies represent an evolving area of research, and future meta-analyses incorporating these treatments will further refine the understanding of safety profiles in AD.

In conclusion, this extensive meta-analysis examined the ADR patterns of AD drugs by showing substantial changes in the occurrence rates, impact and tolerance capabilities between various drug classes. MCAs acting against amyloid-beta proteins including bapineuzumab, aducanumab and gantenerumab caused numerous ARIA that lead to higher patient withdrawal from clinical trials. AChI (downpezil, rivastigmine, and galantamine) presented better safety results, since adverse drug reactions mostly affected gastrointestinal and nervous systems, but left fewer patients to discontinue treatment. This study reveals new safety risks with atabecestat and other BACE1 inhibitors because they caused liver damage and substantial participant withdrawal from clinical testing. The successful use of anti-AD pharmacotherapies in disease management depends on complete safety profile examination during clinical decision-making processes. 

## Data Availability

The datasets generated and/or analyzed during the current study are publicly available at PubMed, Embase, Web of Science and Scopus and ClinicalTrials.gov websites. References of the articles from which data was generated or analyzed during this study are provided in full within this article in reference section (Reference number 13 to 43).
