# Supplementary material for: Evaluating the safety of investigational anti-Alzheimer’s drugs: a comprehensive meta-analysis in the past to current decade
Source: Dement Neuropsychol. 2026 Aug 24;20:e20250472. doi: 10.1590/1980-5764-DN-2025-0472 (PMC13506175; doi:10.1590/1980-5764-DN-2025-0472)
Supplement: Supplementary file 1 [file 1980-5764-dn-20-e20250472-md1.docx]

**Supplementary Material**

**Table S1.** The summary of the data in studies

| **Study** | **Drug Class** | **Drug Name** | **No. of Participants** | **Dosage (mg/day)** | **Treatment Duration** |
| --- | --- | --- | --- | --- | --- |
| Tariot PN et al., 2001^13^ | Acetylcholinesterase inhibitor | Donepezil | 208 | 10 | 24 |
| Ravina et al., 2004^14^ |  |  | 9 | 10 | 10 |
| Imbimbo BP et al., 2000^15^ |  | Eptastigmine | 230 | 12 | 25 |
| Imbimbo BP et al., 1999^16^ |  |  | 327 | 60 | 24 |
| Burns A et al., 2009^17^ |  | Galantamine | 207 | 24 | 24 |
| Knapp MJ et al., 1994^18^ |  | Physostigmine | 663 | 160 | 30 |
| Farlow M et al., 1992^19^ |  | Tacrine | 468 | 80 | 12 |
| Ferrero et al., 2016^20^ | anti-amyloid-beta (A) monoclonal antibody | Aducanumab | 39 | 60 | 11 |
| Salloway et al., 2009^21^ |  | Bapineuzumab | 124 | 2 | 44 |
| Salloway et al., 2025^22^ |  | Gantenerumab | 993 | 1600 | 116 |
| Van Gool et al., 2001^23^ | anti-inflammatory | Hydroxychloroquine | 168 | 400 | 72 |
| Olin et al., 2001^24^ | Antipsychotics | Carbamazepine | 9 | 400 | 6 |
| Banerjee et al., 2023a^25^ |  |  | 40 | 300 | 12 |
| Banerjee et al., 2023^26^ |  | Mirtazapine | 102 | 45 | 12 |
| Frölich et al., 2011^27^ | CNS-specific NNR agonist | AZD3480 | 244 | 100 | 12 |
| Scheltens et al., 2018^28^ | glutaminyl cyclase inhibitor | PQ912 | 80 | 800 | 12 |
| Craft et al., 2012^29^ | Hormones | Insulin | 74 | 40 | 16 |
| Mulnard et al., 2000^30^ |  | Estrogen | 81 | 1.25 | 52 |
| Teng et al., 2022^31^ | humanized IgG4 monoclonal an- tibody | Semorinemab | 311 | 8100 | 73 |
| Ostrowitzki et al., 2022^32^ |  | Crenezumab | 811 | 60 | 100 |
| Dodel et al., 2013^33^ |  | Octagam® 10 | 40 | 0.8 | 4 |
| Wessels et al., 2020^34^ | Human beta amyloid precursor protein–cleaving enzyme 1 Inhibitor | Lanabecestat | 2638 | 50 | 104 |
| Aisen et al., 2010^35^ | Inhibitor of amyloid aggregation | Tramiprosate | 699 | 150 | 78 |
| Ayton et al., 2025^36^ | Iron chelator | Deferiprone | 53 | 15 | 52 |
| Delnomdedieu et al., 2016^37^ | Monoclonal antibody | AAB-003 | 60 | 8100 | 13 |
| Salloway et al., 2014^38^ |  | Bapineuzumab | 1480 | 2 | 78 |
| Doody et al., 2014^39^ |  | Solanezumab | 1027 | 400 | 72 |
| Novak et al., 2020^40^ | Oral BACE1 inhibitor | Atabecestat | 75 | 50 | 24 |
| Geldmacher DS et al., 2011^41^ | PPARγ agonist | Pioglitazone | 29 | 45 | 72 |
| Wilcock GK et al., 2008^42^ | Selective Aβ42-lowering agent | Tarenflurbil | 139 | 800 | 52 |
| Nappi G et al., 1997^43^ | Vasodilator | Nicergoline | 54 | 30 | 52 |

**Table S2.** ADR Data of drugs across all the drug classes

| **Study** | **Drug Name** | **ADR Type** | **Incidence Rate (%)** | **ADR Count (Test)** | **ADR Count (Control)** |
| --- | --- | --- | --- | --- | --- |
| Aisen et al., 2010^35^ | Tramiprosate | Cardiac arrest, CHF, pneumonia | - | 48 | 26 |
| Ayton et al., 2025^36^ | Deferiprone | Neutropenia | 7.5 | 4 | 0 |
| Banerjee et al., 2023^26^ | Mirtazapine | Pneumonia, cardiac arrest, CKD, and fractures | 66 | 225 | 192 |
| Banerjee et al., 2023a^25^ | Carbamazepine | Gastric hemorrhage | 68 | 106 | 192 |
| Burns A et al., 2009^17^ | Galantamine | Mild to moderate adverse events | 88 | 183 | 177 |
| Craft et al., 2012^29^ | Insulin | Mild rhinitis | 5 | 106 | 27 |
| Delnomdedieu et al., 2016^37^ | AAB-003 | Amyloid-related imaging abnormalities (ARIA-H) | 12.5 | 42 | 12 |
| Dodel et al., 2013^33^ | Octagam® 10 | ARIA-H | 59.5 | 37 | 14 |
| Doody et al., 2014^39^ | Solanezumab | Cardiac problems | 3.5 | 72 | 80 |
| Farlow M et al., 1992^19^ | Tacrine | Liver toxicity, nausea, vomiting, diarrhea | 25 | 117 | 78 |
| Ferrero et al., 2016^20^ | Aducanumab | Headache, diarrhea, and URI | 54 | 21 | 5 |
| Frölich et al., 2011^27^ | AZD3480 | Gastrointestinal-related adverse events | 40.5 | 78 | 60 |
| Geldmacher DS et al., 2011^41^ | Pioglitazone | Peripheral edema | 28.6 | 11 | 6 |
| Imbimbo BP et al., 1999^16^ | Eptastigmine | Gastrointestinal events | 54 | 115 | 89 |
| Imbimbo BP et al., 2000^15^ | Eptastigmine | Nausea, vomiting, diarrhea, abdominal pain | 52 | 89 | 55 |
| Knapp MJ et al., 1994^18^ | Physostigmine | Liver toxicity, gastrointestinal issues | 28 | 187 | 120 |
| Mulnard et al., 2000^30^ | Estrogen | Vaginal bleeding and DVT | - | 14 | 2 |
| Nappi G et al., 1997^43^ | Nicergoline | No significant adverse events | 7 | 4 | 1 |
| Novak et al., 2020^40^ | Atabecestat | Hepatic-related AEs | 71.1 | 18 | 5 |
| Olin et al., 2001^24^ | Carbamazepine | Diarrhea and vomiting | 45 | 13 | 18 |
| Ostrowitzki et al., 2022^32^ | Crenezumab | Headache, URI, back pain, Nasopharyngitis | 79.7 | 644 | 628 |
| Ravina et al., 2004^14^ | Donepezil | Worsening psychosis and arrhythmia | 52 | 11 | 9 |
| Salloway et al., 2009^21^ | Bapineuzumab | Unspecified ADR | 94 | 37 | 20 |
| Salloway et al., 2014^38^ | Bapineuzumab | Amyloid-related imaging abnormalities with effusion or edema | 92.6 | 44 | 27 |
| Salloway et al., 2025^22^ | Gantenerumab | ARIA-H | 22 | 235 | 98 |
| Scheltens et al., 2018^28^ | PQ912 | Gastrointestinal and skin-related issues | - | 135 | 103 |
| Tariot PN et al., 2001^13^ | Donepezil | Gastrointestinal events, weight loss | 97 | 97 | 96 |
| Teng et al., 2022^31^ | Semorinemab | Headache, URI, back pain, Nasopharyngitis | 91.6 | 286 | 121 |
| Van Gool et al., 2001^23^ | Hydroxychloroquine | Severe adverse event | - | 13 | 0 |
| Wessels et al., 2020^34^ | Lanabecestat | Psychiatric adverse events, weight loss, and hair color changes | 89.5 | 360 | 158 |
| Wilcock GK et al., 2008^42^ | Tarenflurbil | Diarrhea, nausea, dizziness | - | 44 | 13 |
